# Concurrent Change in Serum Cholinesterase Activity and Midregional-Proadrennomedullin Level Could Predict Patient Outcome following Liver Transplantation

**DOI:** 10.3390/biom12070989

**Published:** 2022-07-15

**Authors:** Sebastian O. Decker, Albert Krüger, Henryk Wilk, Florian Uhle, Thomas Bruckner, Stefan Hofer, Markus A. Weigand, Thorsten Brenner, Aleksandar R. Zivkovic

**Affiliations:** 1 Department of Anesthesiology, Heidelberg University Hospital, Im Neuenheimer Feld 420, 69120 Heidelberg, Germany; albert.krueger@gmx.net (A.K.); henryk.wilk@med.uni-heidelberg.de (H.W.); florian.uhle@med.uni-heidelberg.de (F.U.); markus.weigand@med.uni-heidelberg.de (M.A.W.); thorsten.brenner@uk-essen.de (T.B.); 2 Institute of Medical Biometry and Informatics, Heidelberg University, Im Neuenheimer Feld 130, 69120 Heidelberg, Germany; bruckner@imbi.uni-heidelberg.de; 3 Department of Anesthesiology, Westpfalzklinikum, Kaiserslautern, Hellmut-Hartert-Straße 1, 67655 Kaiserslautern, Germany; shofer@westpfalz-klinikum.de; 4 Department of Anesthesiology and Intensive Care Medicine, University Hospital Essen, University Duisburg-Essen, Hufelandstr. 55, 45147 Essen, Germany

**Keywords:** liver transplantation, pseudocholinesterase, outcome, mid-regional proadrenomedullin

## Abstract

Background: After liver transplantation (LTX), patients are susceptible to opportunistic infections resulting in reduced outcomes within the early post-transplantation period. The postoperative monitoring of LTX patients has gained much importance in recent years. However, reliable plasmatic markers predicting 90-day outcomes are still lacking. Methods: In the post hoc analysis of a prospective, observational study, butyrylcholinesterase (BChE), mid-regional proadrenomedullin (MR-proADM), as well as conventional inflammatory markers (procalcitonin, C-reactive protein) were evaluated in 93 patients at seven consecutive timepoints within the first 28 days following LTX. Results: Persistently reduced activity of BChE and elevated MR-proADM levels indicated reduced 90-day survival following LTX. Furthermore, reduced BChE and increased MR-proADM activity could indicate early post-transplantation bacterial infections, whereas conventional inflammatory biomarkers showed no diagnostic efficacy within the observation period. Conclusion: Concurrent assessment of BChE and MR-proADM activity might serve as a bedside diagnostic tool for early bacterial infections following liver transplantation. Thus, a combined utilization of the two biomarkers may be a useful tool in the risk evaluation of patients following liver transplantation.

## 1. Introduction

Liver transplantation (LTX) is an established therapeutic method for patients suffering from end-stage liver disease [1]. Although the outcome of transplanted patients has significantly improved, severe infections, acute transplant rejection, biliary complications, vascular complications, and early allograft dysfunction remain major postoperative complications. These events may cause the failure of the transplanted organ and lead to worsening outcomes or death [2,3,4].

The highest incidence of opportunistic infections caused by bacteria has been observed within the first month following LTX and ranges between 30% and 75%, depending on the reporting center [5,6,7,8]. However, early and reliable diagnosis of bacterial infections remains a challenge. Clinical signs such as fever or leukocytosis may be absent, resulting in severe complications caused by minor infections [9]. In addition to culture-based diagnostic methods for the detection of bacterial infections, plasmatic infection biomarkers such as C-reactive protein (CRP), procalcitonin (PCT), and interleukin (IL)-6 have long been established in clinical practice. Nevertheless, these biomarkers show considerable limitations [10,11,12].

Immunosuppression causes an inadequate immune response to pathogens. Suppressed immune reaction might result in the undetected escalation of infection, leading to severe sepsis, organ failure, and death. The cholinergic anti-inflammatory pathway, a neuro-immune regulatory mechanism, has been shown to be effective in modulating the immune response in an animal model [13,14,15] and in humans [16,17]. Butyrylcholinesterase (BChE) is an enzyme abundant in blood. BChE is synthesized in the liver and has been routinely used as a liver function test. BChE hydrolyzes acetylcholine, rendering this enzyme a putative modulator of non-neuronal cholinergic activity [18]. Indeed, a reduction in BChE activity has been associated with the onset of sterile inflammation [19]. Moreover, sustained reduction of BChE activity correlated with poor outcomes in septic patients [20,21]. Nevertheless, the aforementioned change in BChE activity could not be used to identify the nature of the inflammation.

Vasodilatory hormone adrenomedullin (ADM), synthesized in multiple locations in the human body such as the heart, lung, kidney, and blood vessels, plays an important role in the inflammatory response. The synthesis of ADM depends on a two-step conversion, where the prehormone preporadrenomedullin is cleaved to proadrenomedullin and finally to ADM. MR-proADM occurs as a side product in a 1:1 ratio and may therefore be used for biochemical analysis of ADM levels [22]. Increased levels of MR-proADM are described in several diseases, such as heart failure, liver cirrhosis, and renal failure, but also in severe infections and sepsis [22]. Moreover, increased plasmatic levels of MR-proADM are shown to be indicative of poor outcomes, in particular when measured in patients with septic shock and in those with acute heart failure [23,24].

Here, we assessed a quick, sensitive, and specific diagnostic approach for the detection of early bacterial infections following liver transplantation. We measured the enzymatic activity of BChE, a highly sensitive indicator of inflammatory response. Furthermore, these analyses were complemented by measuring serum levels of the inflammatory biomarker MR-proADM. Finally, we analyzed the efficacy of the novel diagnostic approach to predict the outcome of the patient following liver transplantation.

## 2. Materials and Methods

### 2.1. Study Design

**Figure 1:**
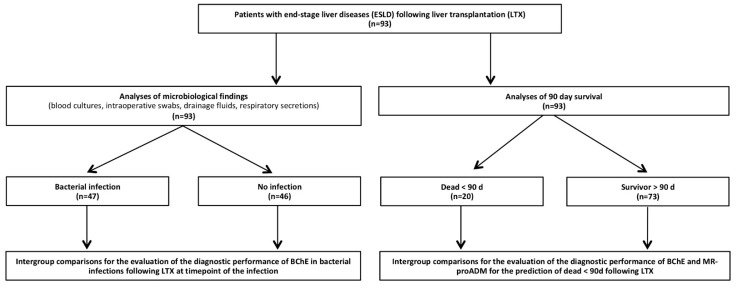
Flowchart of included patients. Abbreviations: ESLD, end-stage liver disease; LTX, liver transplantation, BChE, butyrylcholinesterase; MR-proADM, mid-regional proadrenomedullin.

This study represents post hoc analysis of the data obtained for a previously published observational study powered for fungal infections in patients following LTX [25]. The study was approved by the local ethics committee (Ethics Committee of the Medical Faculty of Heidelberg, Trial Codes No. S-098/2013/German Clinical Trials Register: DRKS00005480). Plasma samples and clinical data collection were conducted in the surgical intensive care unit of the Heidelberg University Hospital, Germany, between February 2014 and March 2016. Written informed consent was obtained from all 93 enrolled patients. All included patients suffered from severe chronic liver disease and therefore received an orthotropic, deceased donor LTX. Following transplantation, all patients were treated according to the Heidelberg Manual for Liver Transplantation [26]. This protocol includes a defined immunosuppressive regime, as well as standardized infection prophylaxis by a specialized transplant care team. All blood samples were collected directly after LTX, at day 1 (d1), day 2 (d2), day 7 (d7), day 14 (d14), day 21 (d21), and day 28 (d28) following liver transplantation. The activity of BChE was measured using a point-of-care (POCT) device, as previously described elsewhere [21]. The activity of MR-proADM was measured according to the manufacturer’s specifications. Patient characteristics (age, gender, nature of the liver disease), medication, as well as clinical scores, including a model for end-stage liver disease (MELD) score, simplified acute physiology score (SAPS II), sequential organ failure assessment score (SOFA), and acute physiology health evaluation score (APACHE II) were obtained from patients’ electronic documents. Laboratory parameters (e.g., leukocytes, procalcitonin (PCT), C-reactive protein (CRP)) were obtained according to the standardized central laboratory protocols. A detailed study flowchart is shown in Figure 1. The primary objectives of the study were to examine BChE activity for the prediction of patient survival following LTX. Secondary objectives focused on BChE and MR-proADM regarding the prediction of bacterial infections following LTX. The study was performed according to the Strobe statement (Supplemental Table S1).

### 2.2. Study Group Definitions

Study patients were divided into 90-day survivor and non-survivor groups (Figure 1). Furthermore, patients were screened for bacterial infections according to the criteria of the American Society of Transplantation published in 2006 [27], combined with clinical signs and plasmatic infection parameters, as previously published in [28]. In brief, bacterial infection was suspected if (1) a positive blood culture was accompanied by clinical deterioration (e.g., fever, hypotonia with systolic blood pressure < 90 mmHg), increased plasmatic infection markers (CRP > 10 mg/dL or PCT > 1 µg/L), and requirement for antibiotic treatment, (2) if positive bacterial findings in intraoperatively collected swabs or in fluids from subsequently inserted interventional drainages of primary sterile body cavities (in case of radiologically or surgically diagnosed abscesses) were combined with increased plasmatic infection markers (CRP > 10 mg/dL or PCT > 1 µg/L) and required antibiotic treatment, or (3) if high bacterial burden in existing drainage cultures was accompanied by clinical deterioration (e.g., fever, hypotonia with a systolic blood pressure < 90 mmHg), increased plasmatic infection markers (CRP > 10 mg/dL or PCT > 1 µg/L), and requirement for antibiotic treatment. To normalize the analysis, we introduced a virtual timepoint (V) of the first bacterial infection. Furthermore, infected patients were compared to a comparison group including unobtrusive age- and sex-matched patients.

### 2.3. Statistical Analysis

All data were saved into an electronic database (Excel 2019; Microsoft Corp, Redmond, WA, USA) and evaluated using SPSS software (Version 26.0; SPSS, Inc., Chicago, IL, USA). Figures were generated using Graphpad Prism 8 (GraphPad Software, La Jolla, CA, USA), SPSS software, and assembled with the presentation software PowerPoint 2019 (Microsoft Corp). Categorical data were shown as absolute and relative frequencies. Quantitative data were presented as median with quartiles. The Kolmogorov–Smirnov test was used to check for normal distribution. Due to non-normally distributed data, non-parametric methods for evaluation were used (Chi-square test for categorical data, Mann–Whitney U test for continuous data). Appropriate cut-off values for significant results were calculated using receiver-operating-characteristics (ROC) analyses, whereas the combined biomarker performance was tested with a logistic regression model. Spearman correlation was used to detect significant correlations between plasmatic biomarkers. Confounder assessment for the 90-day survival was performed using a multivariate binary logistic regression model. Kaplan–Meyer curves were drawn to show the differences in patients’ survival. A *p*-value < 0.05 was considered statistically significant. Concerning symbolism and higher orders of significance: *p* < 0.05: *, *p* < 0.01: **, *p* < 0.001: ***.

## 3. Results

### 3.1. Patient Characteristics

Patient characteristics of 90-day survivors and non-survivors are presented in Table 1 and Table 2. Non-survivors required higher catecholamine doses before LTX (Table 1) and revealed higher critical illness scores on the first day following LTX (d1): APACHE-score for non-survivors 29.5 (23.3–34.0) vs. survivors 25.0 (16.0–31.0); SAPS-score for non-survivors 66.5 (46.0–78.0) vs. survivors 44.0 (29.0–67.0). Furthermore, the non-survivor group showed a significantly increased duration of mechanical ventilation (7.0 days (3.3–16.3)) vs. survivors (1.0 days (1.0–2.0)). More patients in the non-surviving group required dialysis after LTX than those in the surviving group (non-survivor *n* = 14 (70%) vs. survivor *n* = 13 (17.8%)). Finally, surgical complications were more prevalent in non-survivors (non-survivor: *n* = 20 (100%) vs. survivors *n* = 24 (32.9%)). Out of the 93 patients, 47 revealed a bacterial infection within the first 28 days following LTX, whereas 46 were unobtrusive. Detailed data regarding the bacterial findings are shown in Supplemental Table S2, whereas causes of death are listed in Table 3.

### 3.2. Reduced BChE Activity Predicts Patient Outcome

**Figure 2:**
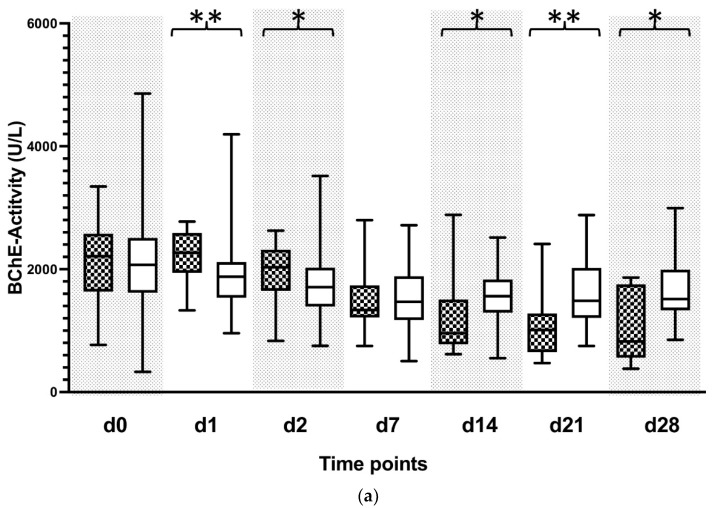

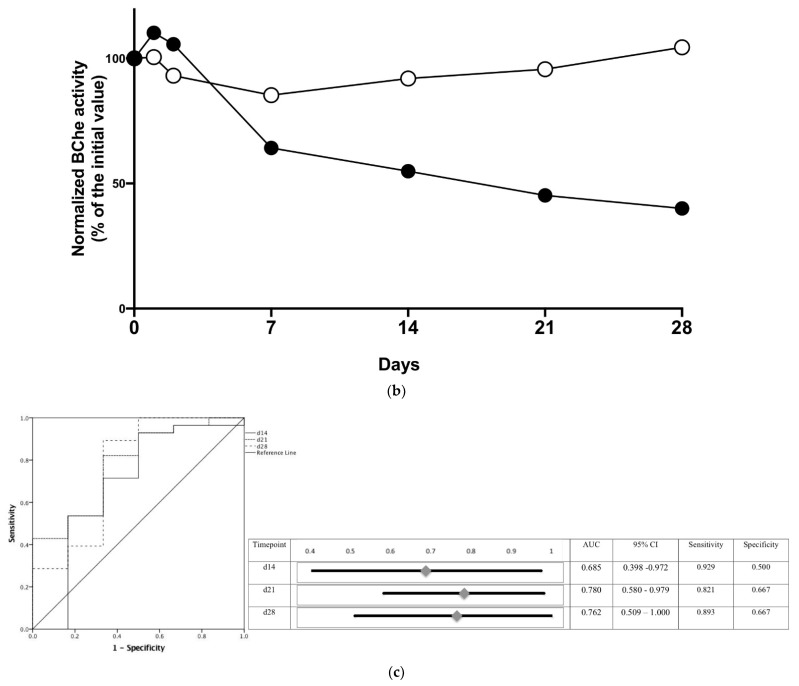

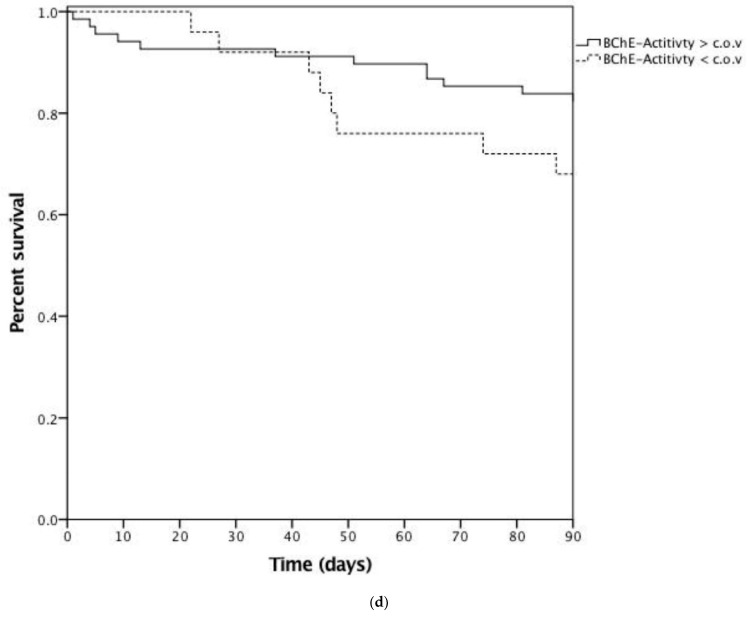
Plasma concentrations of butyrylcholinesterase (BChE) in patients following liver transplantation. (**a**) Plasmatic BChE activity was measured in patients following liver transplantation and grouped according to their 90-day survival. BChE activity is presented in kU/L for 90-day survivors (white box) and 90-day non-survivors (black squared box). Plasma samples were collected immediately following liver transplantation (d0), and on day 1 (d1), day 2 (d2), day 7 (d7), day 14 (d14), day 21 (d21), and day 28 (d28) afterwards. Data in the box plots are presented as the median, 25th percentile, and 75th percentile, with the 10th as well as 90th percentile at the end of the whiskers. Concerning symbolism and higher orders of significance: *p* < 0.05: *, *p* < 0.01: **, Mann–Whitney U Test. (**b**) Plasmatic BChE activity represents normalized values to the initial value at day 0. Values are shown in percentages. (**c**) Receiver operating characteristic (ROC) analysis with BChE in all participating patients on day 14 (d14), as well as day 21 (d21) and day 28 (d28), respectively, with regard to the prediction of 90-day survival. Patients who died within 90 days after liver transplantation represented the target group, whereas surviving patients served as controls for this ROC analysis. (**d**) A comparison of Kaplan–Meier curves stratified according to the cut-off-value (c.o.v.) calculated in (**b**) for the timepoint d14.

Enzymatic activity of serum cholinesterase showed no difference between the groups when measured upon completion of the LTX procedure (d0). Non-survivors showed higher levels of BChE activities on days 1 (d1) and 2 (d2) following the surgery compared to the survivor group. A sustained reduction of the BChE activity was observed in the non-surviving group starting on day 7 following transplantation. Moreover, measurements of the BChE activity on days 7 through 28 remained consistently lower in the non-surviving group as compared to the survivors (Figure 2a). To counteract the observed inter-individual variability, BChE activities were normalized to the baseline value (d0). Starting on day 7 following the procedure (d7), normalized BChE activity showed a sustained reduction in the non-surviving group. Initially observed reduction in the normalized BChE activity recovered during the observation period in the surviving patients (Figure 2b). The diagnostic accuracy for the BChE enzymatic activity to predict 90-day patient outcome was evaluated with the ROC analysis. Fourteen days following the procedure (d14), the prediction accuracy for the BChE activity was 0.685 (0.398–0.972) (area under the curve with a 95% confidence interval (CI)). Furthermore, prediction accuracy assessed 21 days following the surgery (d21) revealed an AUC (with 95% CI) of 0.780 (0.580–0.979). Lastly, prediction accuracy on the 28th day following transplantation (d28) revealed an AUC (with 95% CI) of 0.762 (0.509–1.000) (Figure 2c).

Finally, patients with a BChE-activity lower than 1029.5 U/L showed reduced 90-day survival (Figure 2d).

BChE is an enzyme synthesized in the liver and abundantly present in serum. We tested whether intraoperative transfusion of blood components might affect the level of the BChE enzymatic activity and patient outcome. Non-surviving patients received more intraoperative transfusions compared to survivors (5 (1.5–8.0) bags of packed red cells in non-survivors and 2 (0–4.75) in survivors; *p* = 0.011 *, 10 (6.5–19) bags of fresh frozen plasma in non-survivors and 8 (0.75–16.5) in survivors); *p* = 0.535). Moreover, multivariate regression analysis showed that intraoperative transfusion of blood products did not affect the 90-day outcome in patients subjected to liver transplantation (Supplemental Table S3). A functional liver is a prerequisite for BChE synthesis. Therefore, we tested whether organ function or the lack thereof, might influence the levels of BChE enzymatic activity. Median plasmatic liver enzyme concentrations decreased to normal ranges within the first week following LTX in both surviving and non-surviving patients. Furthermore, no differences between survivors and non-survivors were observed regarding alanine-aminotransferase (ALAT) and aspartate-aminotransferase (ASAT) activities nor in plasmatic coagulation parameters (Supplemental Table S4).

### 3.3. Reduced BChE Activity Is Associated with Systemic Inflammation

**Figure 3:**
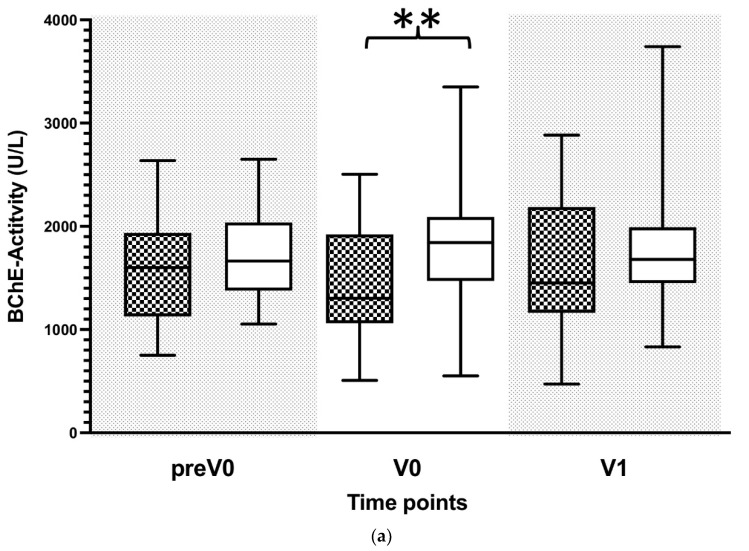

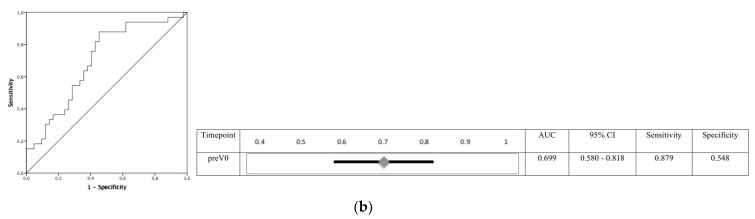
Plasma concentrations of butyrylcholinesterase (BChE) in patients following liver transplantation adjusted to the timepoint of bacterial infection. (**a**) Plasmatic BChE activity was measured in patients following liver transplantation. Measurements in patients with bacterial infection were adjusted to the timepoint of appearance (black squared box) and then compared to non-infected patients (white box). In patients with a bacterial infection, new timepoints were assigned by matching them to the first time of bacterial infection, whereas the control group without bacterial infection was assigned by matching them in an age- and sex-related manner to the same timepoints as patients with a bacterial infection. The following virtual timepoints were created: the first measurement before the first bacterial infection (preV0), the plasma level at the time of bacterial infection (V0) and the next measured plasma level (V1). BChE activity is presented in kU/L. Data in box plots are given as median, 25th percentile, and 75th percentile, with the 10th and 90th percentile at the end of the whiskers. Concerning symbolism and higher orders of significance: *p* < 0.01: **, Mann–Whitney U Test. (**b**) Receiver operator characteristics (ROC)-analysis for BChE in bacterial infected vs. uninfected patients regarding the virtual timepoint V0 that is described above. Abbreviations: AUC, area under the curve; CI, confidence interval.

Reduced BChE activity was observed in patients with systemic inflammation. Here, we tested whether reduced BChE activity correlates with bacterial infections in transplanted patients. After identifying the presence of bacterial infection, we measured the BChE activity in transplanted patients and compared these values to those obtained from the matched unobtrusive transplanted patients. Indeed, BChE activity was significantly lower in patients with a bacterial infection as compared to the matched unobtrusive patient group (Figure 3a). A subsequent ROC analysis revealed an AUC of 0.699 with the best cut-off value of 1.346 kU/L, suggesting a bacterial infection in patients following LTX (Figure 3b).

### 3.4. Increased MR-ProADM Activity in Blood Indicates a Commencing Bacterial Infection in Transplanted Patients

**Figure 4:**
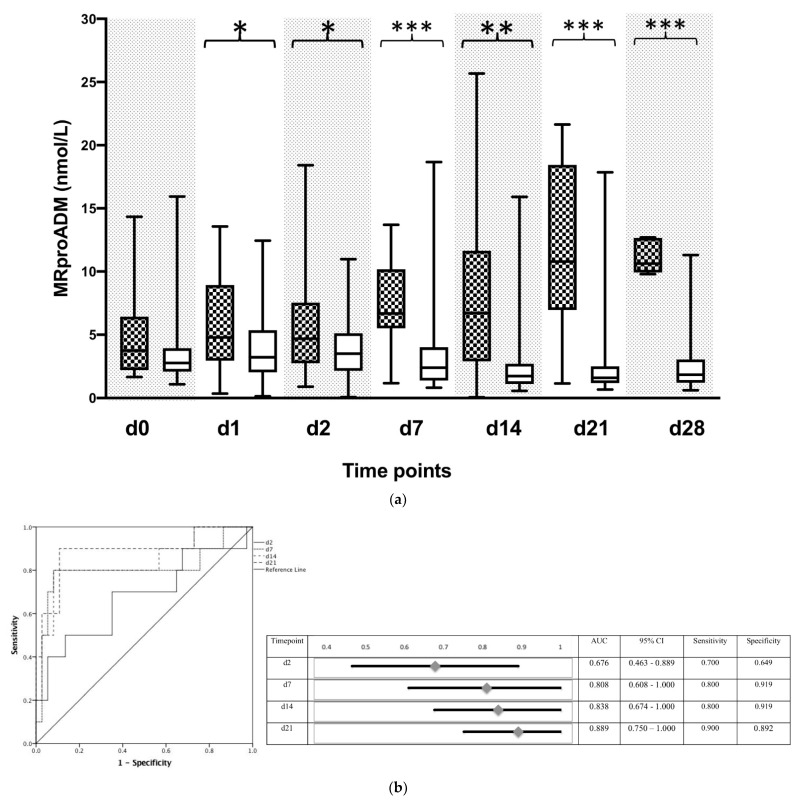
Plasma concentrations of mid-regional proadrenomedullin (MR-proADM) in patients following liver transplantation. (**a**) Plasma concentrations of MR-proADM were measured in patients following liver transplantation and divided according to their 90 d survival. MR-proADM is presented in nmol/L for 90-day survivors (white box) and 90-day non-survivors (black squared box). Plasma samples were collected immediately following liver transplantation (d0), and on day 1 (d1), day 2 (d2), day 7 (d7), day 14 (d14), day 21 (d21), and day 28 (d28) afterwards. Data in box plots are given as median, 25th percentile, and 75th percentile, with the 10th and 90th percentile at the end of the whiskers. Concerning symbolism and higher orders of significance: *p* < 0.05: *, *p* < 0.01: **, *p* < 0.001: ***. Mann–Whitney U Test. (**b**) Receiver operating characteristic (ROC) analysis with MR-proADM in all participating patients at day 2 (d2), as well as day 7 (d7), day 14, and day 21 (d21) afterwards with regard to the prediction of 90-day survival. Patients who died within 90 days after liver transplantation represented the target group, whereas surviving patients served as controls for this ROC analysis.

Reduced BChE activity identifies systemic inflammation with high sensitivity. Nevertheless, the activity change of this enzyme fails to identify the origin of the inflammatory response. Moreover, the induction of a mandatory immunosuppressive regime further complicates the interpretation of the conventional inflammatory biomarkers in transplanted patients. We tested whether the activity change of the novel inflammatory biomarker MR-proADM could indicate a commencing bacterial infection in transplanted and immunosuppressed patients. Starting from day 1 following surgery, plasma levels of MR-proADM were significantly higher in non-surviving patients as compared to the surviving group (Figure 4a). Furthermore, the accuracy of MR-proADM to predict the 90-day survival of transplanted patients revealed an AUC of 0.676 (95% CI: 0.463–0.889) with the best cut-off value of 4.75 mmol/L on postoperative day 2, followed by an AUC of 0.808 (95% CI: 0.608–1.000), with the best cut-off value of 6.35 mmol/L on postoperative day 7 and an AUC of 0.838 (95% CI: 0.674–1.000) with the best cut-off value of 6.29 mmol/L on day 14 following transplantation procedure (Figure 4b).

**Figure 5:**
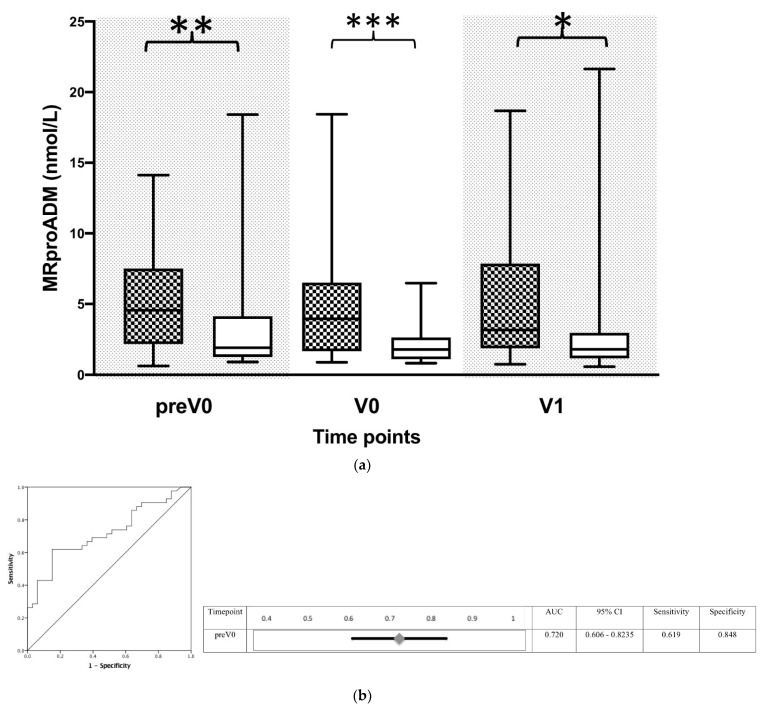
Plasma concentrations of mid-regional proadrenomedullin (MR-proADM) in patients following liver transplantation adjusted to the time of bacterial infection. (**a**) Plasma concentrations of MR-proADM were measured in patients following liver transplantation. Measurements in patients with bacterial infection were adjusted to the time of appearance (black squared box) and then compared to non-infected patients (white box). In patients with a bacterial infection, new timepoints were created by matching them to the first occurrence of bacterial infection, whereas the control group without bacterial infection was built by matching them in an age- and sex-related manner to the same timepoints as those for patients with bacterial infection. The following virtual timepoints were created: the first measurement before the first bacterial infection (preV0), the plasma level at the time of bacterial infection (V0) and the subsequent measured plasma level (V1). MR-proADM is presented in nmol/L. Data in box plots are given as the median, 25th percentile, and 75th percentile, with the 10th and 90th percentile at the end of the whiskers. Concerning symbolism and higher orders of significance: *p* < 0.05: *, *p* < 0.01: **, *p* < 0.001: ***. Mann–Whitney U Test. (**b**) Receiver operator characteristics (ROC)-analysis for MR-proADM in bacterial infected vs. uninfected patients regarding the virtual timepoint V0, described above. Abbreviations: AUC, area under the curve; CI, confidence interval.

Moreover, MR-proADM levels were significantly increased when measured at the time point of the initial diagnosis (e.g., the first bacterial findings) in transplanted patients (Figure 5a). Predicting the accuracy of MR-proADM for identifying a bacterial infection following liver transplantation revealed an AUC of 0.720 (95% 0.606–0.823) with the best cut-off value of 2.90 mmol/L ROC Analysis, Figure 5b).

Finally, we tested whether a concurrent measurement of BChE and MR-proADM might be advantageous in predicting a 90-day survival of transplanted patients. Indeed, the combined use of BChE and MR-proADM measurements revealed the best predicting accuracy with an AUC of 0.996 (95% CI: 0.988–1.000) seven days following LTX. Combined use of BChE and MR-proADM for the detection of a systemic inflammation caused by bacterial infection revealed an AUC of 0.762 with a 95% CI of 0.649–0.875.

In addition, we tested whether conventional inflammation biomarkers (e.g., CRP, PCT, IL-6) could effectively identify and predict bacterial infections during the 28-day observation period in patients following liver transplantation. Conventional inflammatory biomarkers did not provide any additional information towards the diagnosis of bacterial infection following liver transplantation (Supplemental Table S5).

## 4. Discussion

Here, we show that the reduced activity of BChE might indicate an inflammatory response in immunosuppressed patients following liver transplantation. Furthermore, increased levels of MR-proADM in these patients were associated with a commencing bacterial infection. One of the most significant findings to emerge from this study is that a concurrent assessment of BChE and MR-proADM activities in the early phase following liver transplantation could predict 90-day patient outcomes.

Several reports have shown that the cholinergic anti-inflammatory response might play an important role in modulating the immune response following a noxious stimulus [19,29]. The non-neuronal cholinergic system has been shown to be involved in sterile as well as infectious inflammation [20,21,29]. Very little was found in the literature on the question of the cholinergic response in immunosuppressed individuals. Prior studies that noted the importance of early diagnostics of bacterial infections following organ transplantation failed to provide an adequate method for sufficient postoperative monitoring of these patients [12,30,31,32].

Patients undergoing liver transplantation are subjected to initial organ conditioning and adaptation. This critical period includes the re-establishment of the organ function, wound healing, and recovery under immunosuppression. Therefore, vigilant postoperative monitoring of organ and system function is of utmost importance. Moreover, the underlying disease might have an influence on the graft as well as patient survival [33,34], which could not be observed within our limited study collective.

Conventional monitoring of immune system activity often lacks to provide sufficient information in immunosuppressed individuals, leaving rather too long a time period between the first clinical sign of the infection and the first results of the obtained microbiologic cultures. Such a time window might prove to be too long in the clinical setting following organ transplantation.

Conventional inflammatory biomarker CRP, synthesized in the liver, has routinely been used to identify an inflammatory reaction. Nevertheless, our study showed that measured CRP concentration could not reliably identify bacterial infection. A possible explanation for our finding might be the fact that the rise in circulating concentration of CRP in plasma of immunosuppressed liver transplanted patients took longer in response to inflammation than initially presumed. Indeed, our previous study suggested that the initial CRP elevation was observed 24 h following sterile inflammation in patients undergoing surgical procedures [21]. PCT is a commonly available inflammatory biomarker. Elevated concentration of PCT in serum is associated with an inflammatory stimulus, in particular of bacterial origin, rendering this assay highly specific for indicating bacterial infections in clinical settings [35]. Furthermore, the use of PCT has been shown to be valid in the post-transplantation period [36]. However, in contrast to earlier findings, no predicting power of PCT for new infections in patients following liver transplantation could be detected. The result might partially be explained by longer time intervals used for serial PCT measurements, which were part of the protocol used by our institution.

Concurrent use of two independent assays: one providing an early and sensitive diagnosis of the systemic inflammation (BChE assay), in addition to a rapid and bacterial infection-specific method (MR-proADM assay), might offer additional information for monitoring and therapy of immunosuppressed patients in the critical period following liver transplantation. The initial period following liver transplantation is characterized by transient organ dysfunction [37]. Vigilant monitoring of liver function in the first 24 h following surgery remains an essential part of postoperative intensive care [37,38]. Serum cholinesterase is an enzyme synthesized in the liver [39]. Therefore, it is to assume that the altered BChE concentrations observed in the initial period should be interpreted with caution. Nevertheless, as soon as the transplanted organ function has been re-established, altered BChE activity should no longer solely depend on the liver (as a synthesizing organ) function.

Blood products contain a significant amount of BChE. The presence of this enzyme in packed erythrocyte concentrates and fresh frozen plasma has been shown to be beneficial in the treatment of acute intoxications with organophosphates. Acute and irreversible inhibition of cholinesterases, often causing a life-threatening condition [40], might be treated by the transfusion of red blood cells or fresh frozen plasma (FFP) [41,42]. The therapeutic effect could be explained by increasing plasmatic BChE concentration through the transfusion of red blood cells or FFP [43]. Our findings support this hypothesis. BChE activity measured in non-survivors immediately following the procedure was significantly higher than that measured in the survivors. Indeed, non-surviving patients required more perioperative blood transfusion. Furthermore, the half-life time of BChE has been described to vary between 12 and 14 days [44,45]. In fact, our non-surviving patients showed significantly lower BChE activity levels, as compared to the survivors, starting from day 14 following the procedure. This observation could not be explained by blood transfusions in the later postoperative period. Nevertheless, these results need to be interpreted with caution.

Our group previously showed that the decreased BChE activity in septic patients could be predictive of a reduced 90-day survival [20]. In the present study, reduced BChE levels after 14 days and at later timepoints following liver transplantation are associated with increased 90-day mortality. Nevertheless, these findings may not be directly comparable to the results obtained in septic patients. In our previous study, we excluded patients with severe liver disease or acute liver failure [20]. Conversely, all patients in the present study suffered from severe preoperative liver disease. However, plasmatic liver enzymes normalize after 7 days following organ transplantation. Thus, patients with re-established organ function following liver transplantation could be interpreted as “normal liver function” patients.

Previous studies have shown that increased MR-proADM levels could be associated with severe complications following liver transplantation (e.g., rejection, infection, biliary complications) [46], in particular with those of vascular origin [47]. Moreover, renal impairment has been shown to be associated with increased levels of MR-proADM following liver transplantation [48,49]. The findings reported here showed that non-surviving patients received more secondary surgical interventions and required renal support more often. These findings might explain the elevated MR-proADM levels in our patients. Nevertheless, the reported results are in line with our previous findings observed in septic patients, where MR-proADM and BChE activity were shown to be efficient outcome predictors [20]. It can therefore be assumed that assessment of BChE activity, as well as MR-proADM levels, might help in the early prediction of patient outcomes following liver transplantation. In particular, implementing a combination of both biomarkers might further improve the predicting power. However, more research on this topic needs to be undertaken before the role of the serum cholinesterase as well as the MR-proADM activities during an immune response is more clearly understood.

The immune response following organ transplantation represents a complex mechanism. Prompt diagnosis of secondary complications, in particular bacterial infections, remain a challenge for the intensive care physician. Conventionally used inflammatory biomarkers often fail to reach the threshold required for sufficient and timely diagnosis of the commencing infection in immunosuppressed patients. Indeed, none of these biomarkers (PCT, CRP) revealed significant differences between patients with or without a bacterial infection in our study. This finding is comparable with previously published findings, showing that using standard infection parameters in patients following LTX might be associated with substantial limitations [10,11,12]. Furthermore, PCT levels were shown to increase directly after liver transplantation without a relevant source of infection, presumably due to a transfer from the donor [50,51]. Therefore, sole interpretation of PCT activity changes in patients following liver transplantation might prove insufficient for the detection of bacterial infections [52]. A previous study described the kinetics of the IL-6, a promising proinflammatory biomarker assay. IL-6 showed an initial peak after transplantation, followed by a rapid decrease in plasma concentrations of transplanted patients, despite the use of immunosuppressive drugs [53]. Indeed, IL-6 has been suggested to be used as a superior diagnostic tool for infectious complications following liver transplantation [12]. Nevertheless, elevated IL-6 activity has been observed in hepatic cell recovery following liver transplantation [54,55] as well as in cases of early allograft dysfunction [56,57], rendering this proinflammatory biomarker rather nonspecific for bacterial infections.

Our findings show that measuring the activity of serum cholinesterase in transplanted patients offers a suitable method for quick and accurate monitoring of the inflammatory response in these patients. In addition, concurrent measurement of MR-proADM could further improve the diagnostic accuracy for bacterial infections in the acute postoperative period following liver transplantation.

Finally, a number of important limitations need to be considered. First, time periods between probe samplings were successively longer at later timepoints. Next, the total number of available samples decreased during the observation period reflecting the number of patients who died. In addition, due to the nature of the procedure (urgency), samples obtained prior to liver transplantation were not available to the authors. Blood products transfused during the surgery as well as those given immediately after the transplantation might affect the measured BChE concentration in plasma. Authors suggest caution in interpreting the results obtained from patients who recently received blood products. Importantly, BChE activity change is not specific for bacterial infections. Thus, interpretation of the obtained results should be interpreted in complement to other available methods. Finally, with small sample size, caution must be applied, as the findings might not be transferable to patients receiving other organ transplantations.

Authors should discuss the results and how they can be interpreted from the perspective of previous studies and the working hypotheses. The findings and their implications should be discussed in the broadest context possible. Future research directions may also be highlighted.

## 5. Conclusions

This study has identified plasmatic BChE activity as an efficient and highly sensitive point-of-care testing tool for the detection of bacterial infections following liver transplantation. The second major finding was that concurrent measurement of MR-proADM in these patients further increased the accuracy for early recognition of a commencing bacterial infection. These assays emerged as reliable predictors of 90-day patient outcomes following liver transplantation. The evidence from this study suggests that BChE activity change and MR-proADM, when used in complement to the conventional inflammatory biomarkers as well as microbiological cultural procedures, could help intensive care physicians in the early and efficient recognition of bacterial infections following liver transplantation, postoperative complications with detrimental consequences such as graft loss, or death. Bedside measurement of BChE activity change, complemented with MR-proADM assay, might prove helpful in the decision-making process in the intensive care unit, where quick clinical decisions are essential for the outcome of high-risk patients.

## Figures and Tables

**Table 1:** Patients’ characteristics.

Parameter	Unit	All Patients (*n* = 93)	90 d Non Survivor (*n* = 20)	90 d Survivor (*n* = 73)	*p* *for Non Survivor* vs. *Survivor*
male		58 (62%)	9 (45%)	49 (67.2%)	0.062
age	(years)	52.0 (42.0–58.0)	52 (39.5–58.3)	52 (45.0–58.0)	0.626
BMI	(kg/m^2^)	25.5 (22.9–29.8)	25.52 (23.2–30.5)	26.26 (22.9–29.5)	0.786
MELD-Score		18.0 (11.0–28.0)	19 (12.8–25.3)	17 (10.0–28.0)	0.797
BAR-Score		8.0 (3.0–13.0)	8 (4.0–12.0)	8 (3.0–130.)	0.825
Causes of liver cirrhosis					
Alcohol		27 (29%)	9 (45%)	18 (24.6%)	0.070
Hepatitis B		6 (6.5%)	1 (5%)	5 (6.8%)	0.617
Hepatitis C		10 (10.8%)	2 (10%)	8 (10.9%)	0.633
HCC		25 (26.9%)	5 (25%)	20 (27.4%)	0.538
PSC		16 (17.2%)	1 (5%)	15 (20.5%)	0.090
PBC		5 (5.4%)	1 (5%)	4 (5.4%)	0.708
NASH		7 (7.5%)	0 (0%)	7 (9.6%)	0.172
Other		20 (21.5%)	6 (30%)	14 (19.2%)	0.226
Need for catecholamines before LTPL		3 (3.2%)	3 (15%)	0 (0%)	0.009 **
NYHA 0-I		90 (96.8%)	18 (90%)	72 (98.6%)	0.116
Diabetes mellitus		18 (19.4%)	4 (20%)	14 (19.2%)	0.579
Arterial hypertension		28 (30.1%)	5 (25%)	23 (31.5%)	0.548
Coronary heart disease		10 (10.8%)	2 (10%)	8 (13.7%)	0.591
Chronic obstructive lung disease		7 (7.5%)	2 (10%)	5 (6.8%)	0.489
Smoker		21 (22.6%)	5 (25%)	15 (20.5%)	0.471
Renal insufficiency		20 (21.5%)	4 (20%)	16 (21.9%)	0.535
Pre-existing ARF		10 (10.8%)	5 (25%)	5 (7.1%)	0.289
Pre-existing thrombosis		18 (19.3%)	8 (40%)	10 (13.7%)	0.132
Neurological disorder		43 (46.2%)	13 (65%)	30 (41.1%)	0.074
High-urgency		32 (34.4%)	4 (20%)	28 (38.4%)	0.101
Re-LTPL		16 (17.2%)	4 (20%)	12 (16.4%)	0.467
Immunosuppressive medication					
Corticosteroids		93 (100%)	23 (100%)	70 (100%)	—
Mycophenolate mofetil		92 (98.6%)	20 (100%)	72 (98.6%)	0.785
Ciclosporin		39 (41.9%)	8 (40%)	31 (42.5%)	0.526
Tacrolimus		54 (58.1%)	12 (60%)	42 (57.5%)	0.526

Data are presented either as number (with the corresponding percentage value) or as median (with accompanying quartiles (Q1–Q3). **Legends:** BMI = Body Mass Index, BAR = Balance of risk score, MELD = Model of endstage liver disease, HCC = hepatocellular carcinoma, PSC = primary sclerosing cholangitis, PBC = Primary Biliary Cirrhosis, NASH = Nonalcoholic Fatty Liver Disease, NYHA = New York Heart Association Score, ARF = acute renal failure, LTPL = liver transplantation. Concerning symbolism and higher orders of significance: *p* < 0.01: **

**Table 2:** Information regarding hospital stay and extensive care unit.

Parameter	Unit	All Patients (*n* = 93)	90 d Non Survivor (*n* = 20)	90 d Survivor (*n* = 73)	*p* *for Non Survivor* vs. *Survivor*
APACHE II^+^		27.0 (17.0–32.0)	29.5 (23.3–34.0)	25.0 (16.0–31.0)	0.071
SOFA^+^		13.0 (7.0–15.0)	13.0 (11.0–17.3)	12.0 (6.0–15.0)	0.017 *
SAPS^+^		52.0 (30.0–69.0)	66.5 (46.0–78.0)	44.0 (29.0–67.0)	0.118
Time of mechanical Ventilation	(days)	1.0 (1.0–4.0)	7.0 (3.3–16.3)	1.0 (1.0–2.0)	<0.001 ***
Tracheostomy		11 (11.8%)	9 (45%)	2 (2.7%)	<0.001 ***
Hospital stay before LTPL	(days)	1.0 (1.0–7.0)	1.0 (1.0–1.0)	1.0 (1.0–7.5)	0.228
ICU stay	(days)	13.0 (8.0–24.0)	31.5 (8.0–46.3)	13.0 (8.0–21.0)	0.163
Hospital stay	(days)	34.0 (25.0–52.0)	43.0 (16.5–67.6)	32.0 (25.0–52.0)	0.793
ALF after LTPL		16 (16.1%)	13 (65%)	3 (4.1%)	<0.001 ***
ARF after LTPL		31 (33.3%)	15 (75%)	16 (21.9%)	<0.001 ***
Dialysis					
Directly after LTPL		6 (6.5%)	4 (20%)	2 (2.7%)	0.018 *
In time course		27 (20%)	14 (70%)	13 (17.8%)	<0.001 ***
BDA		22 (23.7%)	4 (20%)	18 (24.7%)	0.420
Duration of surgery	(min)	347.0 (289.0–405.0)	395.0 (306.3–441.0)	335.0 (289.0–385.0)	0.111
Intraoperative blood loss	(L)	3.0 (1.5–4.4)	2.8 (1.9–7.9)	3 (1.4–4.1)	0.457
Rejection		20 (21.5%)	2 (10%)	18 (24.7%)	0.132
Perforation of the intestine or stomach		4 (4.3%)	3 (15%)	1 (1.4%)	0.03 *
BDA-insufficiency (*n* = 22)		5 (22.7%)	2 (50%)	3 (16.7%)	0.292
Stenosis of the bile duct		9 (9.7%)	2 (10%)	7 (9.6%)	0.620
Leakage of the bile duct		10 (10.8%)	2 (10%)	8 (11%)	0.594
Need for surgical intervention		44 (47.3%)	20 (100%)	24 (32.9%)	<0.001 ***
Vascular complications		13 (14%)	6 (30%)	6 (8.2%)	0.030 *
Need for endoscopic diagnostics		13 (14%)	7 (35%)	6 (8.2%)	0.006 **

Data are presented either as number (with the corresponding percentage value) or as median (with accompanying quartiles (Q1–Q3). **Legends:** APACHE II–Score = Acute Physiology and Chronic Health Evaluation, SOFA = Sequential Organ Failure Assessment score, SAPS = Simplified Acute Physiology Score, ICU = intensive care unit, LTPL = liver transplantation, ARF = acute renal failure, ALF = acute liver failure, BDA = biliodigestive anastomosis ^+^ calculated at the first day after transplantation. Concerning symbolism and higher orders of significance: *p* < 0.05: *, *p* < 0.01: **, *p* < 0.001: ***.

**Table 3:** Details about causes of death in non-surviving patients (double naming feasible).

Cause of Death	Number of Patients (*n* = 20)
Septic shock	14 (70.0%)
Primary graft failure	5 (25.0%)
Mesenterial ischemia	1 (5.0%)
Ischemic graft failure	2 (10.0%)
Myocardial infarction	1 (5.0%)
Cerebral infarction	1 (5.0%)
Systemic mycosis caused by *Rhizopus microsporus*	1 (5.0%)

## Data Availability

The data presented in this study are available on request from the corresponding authors.

## Supplementary Materials

The following supporting information can be downloaded at: https://www.mdpi.com/article/10.3390/biom12070989/s1, Supplemental Table S1: STROBE Statement—a checklist of items that should be included in reports of observational studies; Supplemental Table S2: Details about bacterial findings; Supplemental Table S3: Risk factors for death within 90 days following LTX; Supplemental Table S4: Plasma values of transaminases and coagulation parameters; Supplemental Table S5: Plasma values of the infection parameters adjusted to the timepoint of bacterial infection.
